# Efficient Heparin Recovery from Porcine Intestinal Mucosa Using Zeolite Imidazolate Framework-8

**DOI:** 10.3390/molecules27051670

**Published:** 2022-03-03

**Authors:** Mahmood Karimi Abdolmaleki, Deepak Ganta, Ali Shafiee, Carlo Alberto Velazquez, Devang P. Khambhati

**Affiliations:** 1 Department of Biology and Chemistry, Texas A&M International University, Laredo, TX 78041, USA; carlovelazquez@dusty.tamiu.edu (C.A.V.); devang.khambhati@tamiu.edu (D.P.K.); 2 School of Engineering, Texas A&M International University, Laredo, TX 78041, USA; deepak.ganta@tamiu.edu; 3 Department of Chemistry, Cape Breton University, Sydney, NS B1P 6L2, Canada; ali_shafiee@cbu.ca

**Keywords:** heparin, metal–organic framework, zeolite imidazolate framework-8, kinetic, thermodynamic

## Abstract

Heparin is one of the most valuable active pharmaceutical ingredients, and it is generally isolated from porcine intestinal mucosa. Traditionally, different types of commercial resins are employed as an adsorbent for heparin uptake; however, using new, less expensive adsorbents has attracted more interest in the past few years to enhance the heparin recovery. Zeolite imidazolate framework-8 (ZIF-8), as a metal–organic framework (MOF) with a high surface area, porosity, and good stability at high temperatures, was selected to examine the heparin recovery. In this research, we demonstrate that ZIF-8 can recover up to ~70% (37 mg g^−1^) of heparin from porcine intestinal mucosa. A mechanistic study through kinetic and thermodynamic models on the adsorption revealed appropriate surface conditions for the adsorption of heparin molecules. The effect of different variables such as pH and temperature on heparin adsorption was also studied to optimize the recovery. This study is the first to investigate the usage of MOFs for heparin uptake.

## 1. Introduction

**Scheme 1:**
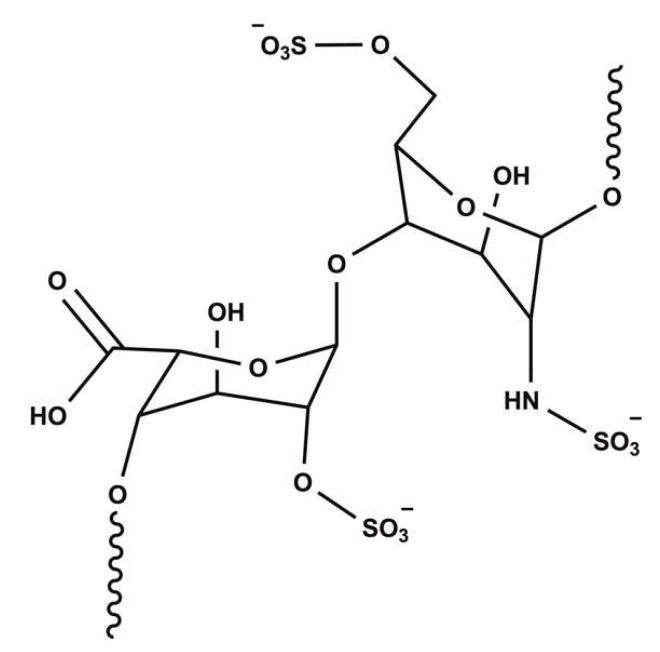
Heparin structure.

Heparin (Scheme 1) is a linear, sulfur-rich polysaccharide in the glycosaminoglycan family with varying lengths and weights ranging from 2000–40,000 kDa; it is generally procured from the bovine and porcine intestinal mucosa. Heparin is predominantly used as an anticoagulant and antithrombotic agent to treat a variety of medical conditions, such as systemic embolism syndrome and deep vein thrombosis; however, it also has applications as an anti-inflammatory and antiviral drug. Furthermore, heparin has been demonstrated to inhibit cancer cell growth and delay the onset of the detrimental symptoms of Alzheimer’s disease [1,2,3,4]. 

Heparin is isolated from porcine intestinal mucosa through a multi-step adsorption/desorption process after digestion has occurred; numerous parameters, namely, adsorbent surface properties, porosity, and thermodynamic and chemical stability, must be considered to securely deliver the highest amount of adsorbed heparin in the final step after elution [5,6,7,8]. In addition, the adsorbent needs to be easily packable (for continuous systems) or easy to separate (for batch systems). To date, several resin beads, such as Amberlite [5], Dowex [8], Lewatit [9], and DEAE [10], have been designed and commercially utilized to recover heparin from porcine intestinal mucosa. Although effective, the methods by which these materials are synthesized are expensive and, in some cases, not environmentally friendly. Recent research has shown the high efficiency and effectiveness of different nanomaterials in the adsorption/desorption of various pharmaceuticals from different matrices [11,12,13,14,15]. Among these nanomaterials, quaternized chitosan/polystyrene microbeads [16], magnetic clay nanotubes [17], cross-linked spherical polycationic [18], and quaternary ammonium-functionalized halloysite nanotubes [19] have especially been designed for the recovery of heparin. 

Metal–organic frameworks (MOFs) are 3D nanoporous materials with an ultra-high surface area (exceeding 6000 m^2^/g), porosity (up to 90% free volume), and thermodynamic/chemical stability. Moreover, their fabrication methods are simple, inexpensive, and environmentally friendly [20,21], making them suitable for commercial use [22,23,24,25,26]. Zeolite imidazole frameworks (ZIFs) are a new class of MOFs consisting of imidazolate linkers and metal ions. Due to their high surface area, pore volume, thermal and chemical stability, and adaptable functionalization, ZIFs have found applications in various fields as sensors, drug delivery agents, gas separation agents, and electronic device components [27,28,29,30,31]. 

In this research, we report the recovery of heparin from the porcine intestinal mucosa using two types of ZIFs (ZIF-8 and ZIF-67) with different surface areas. Our results demonstrated that the ZIF with a higher surface area (ZIF-8) showed a higher amount of heparin recovery. We also evaluated the effects of several variables, such as pH and temperature, to investigate the most optimal conditions for heparin recovery. Our results further illustrated that the heparin recovery with ZIF-8 stabilized after five cycles. We also utilized a sheep plasma test to measure and compare the anticoagulant potency of ZIF-8 with commercial Amberlite FPA98 Cl, the results of which confirmed the significant potential of MOFs in the heparin uptake industry.

## 2. Result and Discussion

### 2.1. ZIF-8 and ZIF-67 Characterization

**Figure 1:**
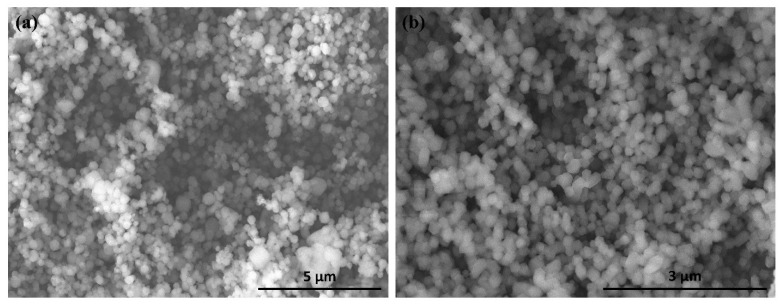
SEM images of (**a**) ZIF-67 (8000×) and (**b**) ZIF-8 (18,000×) nanomaterials.

**Figure 2:**
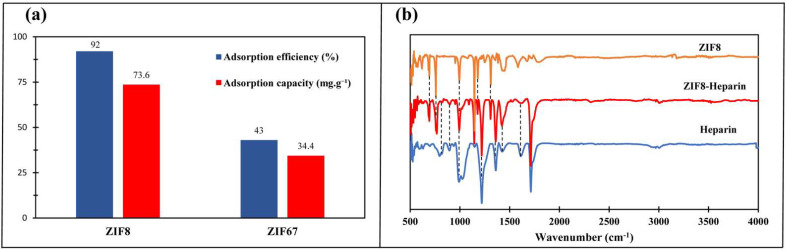
(**a**) Adsorption efficiency of heparin over ZIF-8 vs. ZIF-67 (conditions: 50 mL of 1000 mg L^−1^ heparin standard solution and 0.5 g of the adsorbent); (**b**) FTIR spectra of standard heparin, ZIF-8, and preabsorbed heparin on the ZIF-8.

Scanning electron microscope (SEM) images of ZIF-8 and ZIF-67 were taken at the ACS Materials Facility using a Hitachi S-4800 scanning electron microscope (SEM) at 10 kV. The SEM images are shown in Figure 1. The infrared spectrum of ZIF-8 is shown in Figure 2.

### 2.2. Heparin Adsorption Studies

Prior to optimization, it was important to evaluate the affinity of heparin toward each absorbent in a pure heparin solution. The results (Figure 2a) demonstrated that ZIF-8 could adsorb more than 2-fold more heparin compared to ZIF-67 in a pure aqueous solution. The reported BET surface areas for ZIF-8 and ZIF-67 are >1300 and ~316 m^2^/g, respectively [31,32], which are well correlated with their differences in adsorption efficiency. Due to the superior adsorption capabilities of ZIF-8 over ZIF-67, ZIF-8 was selected for further experiments to evaluate adsorption in real samples containing porcine intestinal mucosa. The FTIR spectrum of ZIF-8 shows a band at 1712 cm^−1^, which confirms the presence of C=N bond stretching. The bands at 1100 cm^−1^ and 990 cm^−1^ are attributed to C-N stretching. Additionally, a preabsorbed sample of ZIF-8 was dried and analyzed via FTIR spectroscopy so as to ensure that heparin adsorption occurred (Figure 2b). The resulting spectrum was compared with the FTIR spectrum of pure sodium heparin salt and the FTIR spectrum of ZIF-8 powder. The appearance of a band at 1712 cm^−1^ in the ZIF-8–heparin mixture is attributed to the C=O group of heparin, and the appearance of a band at 1222 cm^−1^ is attributed to the S=O group of heparin, which confirms the adsorption of heparin on the surface of ZIF-8. 

#### 2.2.1. Effect of pH

**Figure 3:**
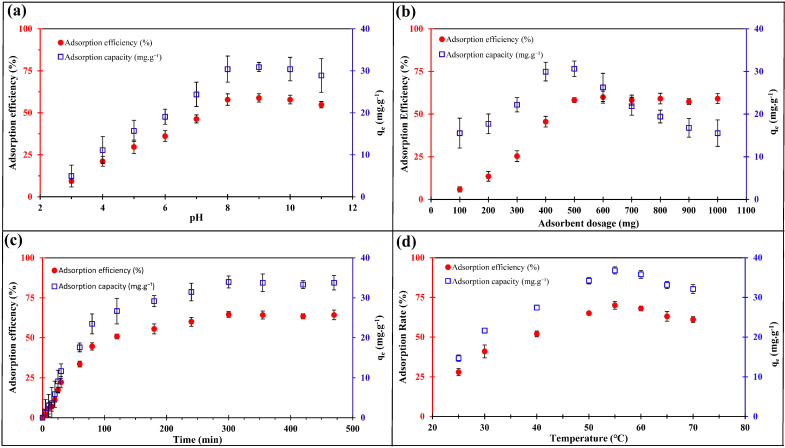
(**a**) Effect of pH (conditions: 50 mL of 1000 mg L^−1^ real sample, 0.5 g of ZIF-8, temperature 50 °C, and 240 min of contact time), (**b**) adsorbent dosage (conditions: 50 mL of 1000 mg L^−1^ real sample at pH = 8, temperature 50 °C, and 240 min of contact time), (**c**) contact time (conditions: 50 mL of 1000 mg L^−1^ real sample at pH = 8, 0.5 g of ZIF-8, and temperature 50 °C), and (**d**) temperature (conditions: 50 mL of 1000 mg L^−1^ real sample at pH = 8, 0.5 g of ZIF-8, and 300 min of contact time) of the adsorption efficiency of heparin over ZIF-8.

One of the most important parameters to consider when studying any adsorption/desorption system is pH, as pH fluctuations can have significant impacts on adsorption efficiency. For this reason, the effect of pH on the adsorption of heparin onto ZIF-8 was investigated by changing the pH of the real sample of the heparin solution from 3 to 11 in a series of experiments; adsorption efficiency and capacity were then evaluated. The results (Figure 3a) indicate that heparin adsorption increases when increasing the pH from 3 to 8. The highest adsorption was observed at a pH of 8, where an efficiency of 57.8% and a capacity of 30.4 mg g^−1^ were recorded; efficiency and capacity subsequently remained constant as more alkaline conditions were induced. Notably, increasing pH slightly deprotonates heparin, resulting in more negative charges that make it more receptive to the positive active sites of ZIF-8. We selected pH = 8 as the optimal pH since the stability of MOFs subsequently decreases at higher pH conditions. 

#### 2.2.2. Effect of the Adsorption Dosage 

ZIF-8 showed a remarkable capacity in the removal of contaminants. In this part of the study, we investigated the effect of the adsorbent dosage of ZIF-8 on heparin uptake by varying the amount of ZIF-8 from 100 to 1000 mg. The results (Figure 3b) indicated that increasing the adsorbent dosage to 500 mg caused the adsorption efficiency and capacity to increase to 58.2% and 30.6 mg g^−1^, respectively. Increasing the dosage of the adsorbent further led to a slight decrease in the capacity while efficiency remained constant. Notably, a higher adsorbent dosage increased the adsorption due to a higher surface area and the presence of more active sites on ZIF-8 adsorbents; the adsorption capacity, however, decreased at dosages higher than 500 mg. This phenomenon is attributed to the interference of other components in the sample, namely, dermatan and chondroitin sulfate, which compete with heparin and prevent it from being adsorbed onto the surface of ZIF-8. Furthermore, the aggregation of ZIF-8 at a higher dosage may also serve to decrease the active sites of the adsorbents for heparin molecules. In addition, the observed decrease in the adsorption capacity from 35 to 15 mg g^−1^ is the result of increasing the mass (m) in Equation (4) [17]. 

#### 2.2.3. Adsorption Time and Temperature Effects

The results from the contact time experiment (Figure 3c) indicated that when increasing the contact time, the adsorption efficiency and capacity increased to 64.5% and 33.9 mg g^−1^, respectively, after 300 min of contact time. This trend was anticipated due to the length of time that it takes heparin molecules to diffuse from the solution and bond to ZIF-8 adsorbent surfaces. As observed in the experiment, adsorption approached stability after that, revealing that ZIF-8 had reached its full capacity at these experimental conditions. In addition to contact time, the solution temperature is another highly influential parameter in adsorption systems. Therefore, to study its effect on heparin uptake, a series of experiments were performed by varying the temperature from 25 °C to 75 °C while keeping all other parameters constant. The results (Figure 3d) demonstrated that by increasing the temperature to 55 °C, the adsorption efficiency and capacity increased to 70% and 36.8 mg g^−1^, respectively. By increasing the temperature, the heparin molecules can move more quickly in the solution due to a decrease in the solution’s viscosity, which facilitates their interaction with the adsorbent surface. The minimal decrease observed after 65 °C is likely due to the decomposition of heparin molecules from temperatures that were too high to support its structural stability. 

#### 2.2.4. Kinetic and Thermodynamic Studies

**Figure 4:**
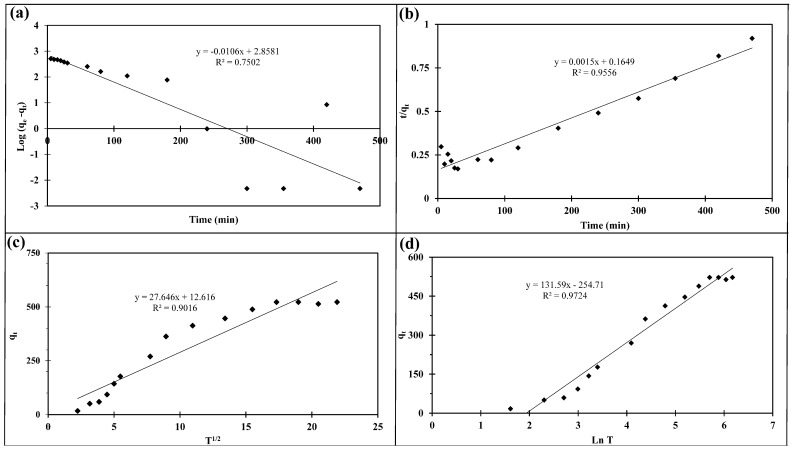
Kinetic study of heparin adsorption over ZIF-8 via (**a**) pseudo-first-order, (**b**) pseudo-second-order, (**c**) intraparticle diffusion, and (**d**) Elovich models.

In order to better comprehend the observed adsorption phenomena of ZIF-8 and its associated mechanisms, the collected data were analyzed via the following kinetic models: pseudo-first-order, pseudo-second-order, intraparticle diffusion, and Elovich (Equations (5)–(8); Figure 4 and Table 1). The obtained data, which are presented in Figure 4 and Table 1, show that the adsorption of heparin onto ZIF-8 best fits the pseudo-second-order kinetic model since the R^2^ value is much closer to 1.0 and q_e(cal)_ is closest to q_e(exp)_ out of all the examined models. Based upon this model, the rate-limiting step is the surface adsorption that involves chemisorption; therefore, the adsorption process is not limited by the concentration of heparin but rather the adsorption capacity of ZIF-8 [32]. This confirms the decrease in heparin uptake with increasing adsorption dosages (Figure 3b). Further, the Elovich model also exhibits a good fit, supporting the adsorption of heparin on the ZIF-8 heterogeneous surface since this model assumes that the rate of the adsorption of the solute decreases exponentially as the amount of adsorption increases.

**Figure 5:**
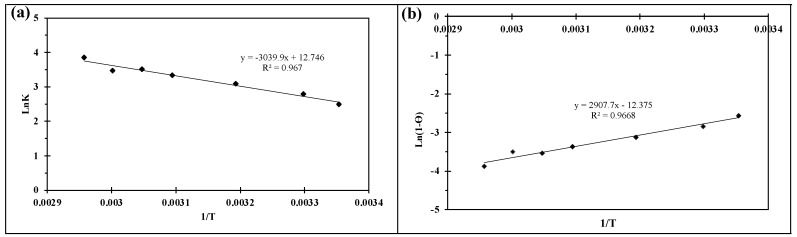
Thermodynamic study of heparin adsorption over ZIF-8 via (**a**) lnK vs. 1/T and (**b**) ln(1 − θ) versus 1/T.

The thermodynamic parameters of heparin adsorption on ZIF-8, including entropy change (ΔS), enthalpy change (ΔH), Gibbs free energy change (ΔG; calculated from ΔG = ΔH − TΔS), and activation energy (Ea), were calculated by using Equations (1) and (2) and plotting lnKc versus 1/T and ln(1 − θ) versus 1/T [33,34]. These data are presented in Figure 5 and Table 2.

(1) $${\mathrm{lnK}}_{c}=\frac{\Delta S}{R}-\frac{\Delta H}{\mathrm{RT}}$$

(2) $$\ln\left(1-\theta \right)={\ln S}^{*}+\frac{E_{a}}{\mathrm{RT}}$$

The positive values of ΔH and ΔS indicate the endothermic nature of the adsorption process and the high affinity of heparin molecules toward ZIF-8. The positive value of E_a_ also further confirms the endothermic nature of the adsorption process, while the negative values of ΔG reflect the feasibility of the process and its spontaneous nature [35]. 

#### 2.2.5. Sorbent Reusability

**Figure 6:**
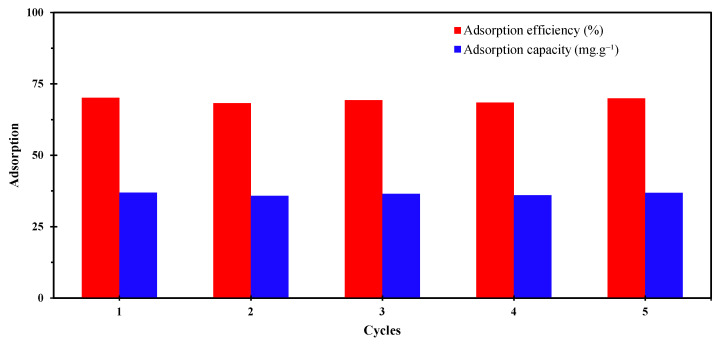
Stability test of heparin recovery over ZIF-8.

ZIF-8 showed impressive performance in heparin uptake in real biological samples, with a ~70% adsorption rate and a 37 mg g^−1^ capacity under optimized conditions. In order to evaluate the industrial potential of ZIF-8, we tested the stability and reusability of ZIF-8 through five adsorption/desorption cycles and harsh regeneration conditions. The results (Figure 6) indicate that the ZIF-8 adsorbent has high stability and reusability even after five adsorption/desorption cycles and exposure to harsh regeneration conditions. 

#### 2.2.6. Sheep Plasma Clotting Assay 

The porcine intestinal mucosa solution used for the sheep plasma clotting assay was prepared via a previously established and reported method [18]. The desired amount of adsorbent was mixed with the mucosa, followed by the stirring of the mixture at 55 °C for 3 h. Lastly, the adsorbent was filtered and washed with Milli-Q water. The solid heparin was then eluted from the adsorbent as described in a previous publication [18]. A similar method was employed for the commercial Amberlite resin. The anticoagulant potency of the recovered heparin from both ZIF-8 and the Amberlite resin was measured and compared [19]. The results indicated that the total activity of the eluted heparin utilizing the ZIF-8 adsorbent was 64 ± 1.8 U per gram of mucosa compared to 59 ± 2.6 U per gram of mucosa from the commercial Amberlite FPA98 Cl resin. These results highlight the promising potential of the ZIF-8 adsorbent in an industrial setting. 

## 3. Experimental Procedures

### 3.1. Materials 

Heparin sodium salt of analytical grade was purchased from VWR, USA. Commercially synthesized ZIF-8 and ZIF-67 were purchased from ACS Material, LLC (Pasadena, CA, USA). Milli-Q water was used for the preparation of all solutions and the adsorption experiments. Porcine intestinal mucosa (heparin content of ~1300 mg L^−1^) was purchased from a local market. The subtilisin enzyme was purchased from STERM Company, Newburyport, MA, USA. Commercial Amberlite FPA98 Cl resin was purchased from Dow Chemical (Midland, MI, USA), USA. Sheep plasma, hydrochloric acid (37%), calcium chloride, analytical grade methanol, sodium hydroxide, and denatured ethanol were purchased from VWR and used as received. Heparin ELISA kits were purchased from MyBiosource, San Diego, CA, USA.

### 3.2. Instrumentation 

A Spectrostar Nano microplate reader (BMG LABTECH, Cary, NC, USA) was used to measure the heparin concentration in both pure and real samples. MOFs, heparin sodium salt, and MOF–heparin were characterized by a Shimadzu IR Affinity FTIR instrument (Shimadzu Scientific Instruments, Columbia, MD, USA). 

### 3.3. Solutions and Methods

A heparin stock solution (1000 ppm) was prepared by adding the required amount of heparin salt to Milli-Q water and used for standard experiments. This solution was used to investigate the adsorption capacity of each adsorbent in the standard condition. For this, 0.5 g of ZIF-8 and ZIF-67 were added to separate solutions, the two mixtures were stirred for 5 h at 55 °C in an incubator, and the adsorbed heparin was calculated for both mixtures by using the methylene blue method according to the previously published procedure [18,19]. Specifically, to examine the material’s absorption capabilities, 250 mg of the MOFs was added to a 25 mL aqueous solution of 1000 ppm heparin sodium. In the incubator, the solution was stirred for 3 h at 55 °C. Five milliliters of the solution was removed and filtered using a syringe filter, and the concentration of heparin in the supernatant was measured using the methylene-blue-assisted spectrophotometric method. Methylene blue (MB) is a cationic metachromatic dye that can bind to heparin. First, 1 mL of the heparin solution was added to 1 mL of MB solution (10 mg L^−1^). Then, the solution was mixed using a vortex mixer for 10 min, and the absorbance was measured by a plate reader based on the intensity of the band at λ_max_ = 663 nm, which is attributed to the free MB. When MB forms a dimer with heparin, the intensity of the band at 663 nm decreases due to the lower concentration of free MB. The concentration of heparin after adsorption was calculated using the pre-plotted standard calibration curve. 

The adsorption efficiency of heparin on the ZIFs and the anticoagulant potency of the eluted heparin were evaluated in porcine intestinal mucosa by using sheep plasma and heparin ELISA kits. The detailed adsorption process can be found in our previous publication [18]. 

The adsorption efficiency and capacity were calculated via Equations (3) and (4):

(3) $$\mathrm{Adsorption}\;\mathrm{efficiency}\;\left(\%\right)=\frac{\left(C_{0}-C_{e}\right)}{C_{0}}\times 100$$

(4) $$\mathrm{Adsorption}\;\mathrm{capacity}\;\left(q_{e}\right)=\frac{\left(C_{0}-C_{e}\right)}{m}\times V$$

where C_0_ and C_e_ (mg L^−1^) are the initial and equilibrium heparin concentrations (measured with the ELISA kit), V (L) is the volume of the mucosa solution that was used for heparin adsorption, and m is the mass of the adsorbent used (g). The experimental conditions of the adsorption were optimized through different influential parameters, such as pH, contact time, and temperature. Furthermore, in order to understand the mechanism of adsorption and also the commercial viability of the adsorbent, other parameters such as adsorbent dosage, kinetic and thermodynamic properties, and the reusability of the absorbent material were investigated. All experiments were carried out in triplicate, and the error bars in the figures indicate the standard deviation of each experiment.

Pseudo-first-order, pseudo-second-order, intraparticle diffusion, and Elovich models were calculated to have a better understanding of the adsorption phenomena. The linearized equation of each model is given in Equations (3)–(6), respectively. The pseudo-first-order model (Equation (5)) considers a proportional relation between the occupation of adsorption sites and the number of unoccupied sites.

(5) $$\log\left(q_{e}-q_{t}\right)={\log q}_{e}-\frac{K_{1}}{2.303}\times t$$

In Equation (5), the value of K_1_ (min^−1^; the rate constant of the second-order equation) and q_e_ can be obtained from the slope and intercept of the linear plot of $\log\left(q_{e}-q_{t}\right)$ versus t, respectively, where q_e_ (mg g^−1^) and q_t_ (mg g^−1^) are the adsorbed heparin at equilibrium and at any time t, respectively [36].

The pseudo-second-order kinetic model assumes that the adsorption rate is not dependent on the heparin adsorption but the adsorption capacity of ZIF-8.

(6) $$\frac{t}{q_{t}}=\frac{1}{K_{2}\times q_{e}{}^{2}}+\frac{t}{q_{e}}$$

In Equation (6), K_2_ (g mg^−1^ min^−1^) and q_e_ (mg g^−1^) are the rate constant of the second-order equation and the maximum adsorption capacity, respectively, which can be calculated from the slope and intercept of the plot of t/q_t_ vs. t [37,38,39].

In the intraparticle diffusion model (Equation (7)), the limiting steps are considered to be mass transport.

(7) qt=Kdiff×t12+C

In this model (Equation (8)), the values of $K_{\mathrm{diff}}$ (the rate constant of intraparticle diffusion (mg g^−1^ min^−1^)) and C (thickness of boundary) are calculated from the slope and intercept of the plot of q_t_ versus t^1/2^ [40]. 

The Elovich kinetic model (Equation (4)) considers the sorbent to have heterogeneous active sites with varying active energies during the adsorption process.

(8) $$q_{t}=\frac{1}{\beta \;\ln\left(\alpha \beta \right)}+\frac{1}{\beta \;\ln\left(t\right)}$$

In this model (Equation (8)), the values of α and β (Elovich constants) can be calculated from the slope and intercept of the plot of q_t_ versus ln(t) [41].

To evaluate the stability of the ZIF-8 adsorbent, after the first adsorption/desorption cycle, ZIF-8 adsorbent was regenerated by washing with a saturated NaCl solution for 3 h at 50 °C following the wash with Milli-Q water. The solution was tested with absorption spectroscopy to ensure that no heparin was carried to the next cycle. The same process was repeated across all five cycles [17].

## 4. Conclusions

Here, we report the use of ZIFs for heparin recovery, which is the first example of heparin recovery utilizing an MOF. To the best of our knowledge, these MOFs have been extensively used for various applications but not for heparin recovery. We employed two different MOFs with different surface areas, and the results met our expectations. ZIF-8, an MOF with a larger surface area than that of ZIF-67, proved to be more effective at adsorbing heparin, with approximately 70% adsorption capacity. Furthermore, our results revealed that the heparin recovered from ZIF-8 had higher total anticoagulant activity compared to the heparin recovered from the commercially available Amberlite FPA98 CI resin (sheep plasma clotting assay). The mechanistic adsorption study demonstrated that the adsorption process adheres to the pseudo-second-order kinetic model and is both thermodynamically endothermic and feasible. We also observed the effects of different variables, such as pH, temperature, and adsorbent dosage, on heparin recovery so as to determine the most optimal conditions for heparin uptake. 

## Figures and Tables

**Table 1:** Calculated parameters from different kinetic study models of heparin adsorption over ZIF-8.

Model	Parameter	Value
Pseudo-first-order	$K_{1}\;\left(\min^{-1}\right)$	0.024
	$q_{e\;\left(\mathrm{cal}\right)}\;\left(\frac{\mathrm{mg}}{g}\right)$	721.31
	$R^{2}$	0.8502
Pseudo-second-order	$K_{2}\;\left(\frac{g}{\mathrm{mg}\;\min}\right)$	1.34 × 10^−5^
	$q_{e\;\left(\mathrm{cal}\right)}\;\left(\frac{\mathrm{mg}}{g}\right)$	672.22
	$R^{2}$	0.9556
Intraparticle Diffusion	$K_{\mathrm{diff}}\;\left(\frac{\mathrm{mg}}{{g\;\min}^{0.5}}\right)$	27.64
	$C\;\left(\frac{\mathrm{mg}}{g}\right)$	12.61
	$R^{2}$	0.901
Elovich	$\beta \;\left(\frac{g}{\mathrm{mg}}\right)$	0.0076
	$\alpha \;\left(\frac{\mathrm{mg}}{g\;\min}\right)$	1.58
	$R^{2}$	0.9724
	$q_{e\;\left(\exp\right)}$	522.11

**Table 2:** Calculated thermodynamic parameters of heparin adsorption over ZIF-8.

**ΔH° (J/mol·K)**	**ΔS°** **(KJ/mol)**	**E**_**a**_ **(KJ/mol)**	**Temperature (K)**						
			298	303	313	323	328	333	338
25.27	105.97	19.41	**ΔG° (KJ/mol)**						
			−31.57	−32.10	−33.16	−34.22	−34.74	−35.27	−35.80

## References

[1] Rodriguez-Torres M.D.P., Acosta-Torres L.S., Díaz-Torres L.A. (2018). Heparin-based nanoparticles: An overview of their applications. J. Nanomater..

[2] Scott L.J., Perry C.M. (2000). Tramadol: A review of its use in perioperative pain. Drugs.

[3] Turpie A.G.G., Hirsh J. (1979). Heparin. Nova Scotia Med. Bull..

[4] Urbinati C., Milanesi M., Lauro N., Bertelli C., David G., D’Ursi P., Rusnati M., Chiodelli P. (2021). HIV-1 tat and heparan sulfate proteoglycans orchestrate the setup of in cis and in trans cell-surface interactions functional to lymphocyte trans-endothelial migration. Molecules.

[5] Vreeburg J.-W., Baauw A. (2010). Method for Preparation of Heparin from Mucosa. Patent.

[6] Lee M.S., Kong J. (2015). Heparin: Physiology, pharmacology, and clinical application. Rev. Cardiovasc. Med..

[7] Anderson J.A.M., Saenko E.L. (2002). Heparin resistance. Br. J. Anaesth..

[8] Flengsrud R., Larsen M.L., Ødegaard O.R. (2010). Purification, characterization and in vivo studies of salmon heparin. Thromb. Res..

[9] Linhardt R.J., Ampofo S.A., Fareed J., Hoppensteadt D., Mulliken J.B., Folkman J. (1992). Isolation and characterization of human heparin. Biochemistry.

[10] Hoke D.E., Carson D.D., Höök M. (2003). A heparin binding synthetic peptide from human HIP/RPL29 fails to specifically differentiate between anticoagulantly active and inactive species of heparin. J. Negat. Results Biomed..

[11] Shafiee A., Aibaghi B., Zhang X. (2021). Reduced graphene oxide-cadmium sulfide quantum dots nanocomposite based dispersive solid phase microextraction for ultra-trace determination of carbamazepine and phenobarbital. J. Braz. Chem. Soc..

[12] Shafiee A., Aibaghi B., Zhang X. (2020). Determination of ethambutol in biological samples using graphene oxide based dispersive solid-phase microextraction followed by ion mobility spectrometry. Int. J. Ion Mobil. Spectrom..

[13] Reddy D.H.K., Yun Y.-S. (2016). Spinel ferrite magnetic adsorbents: Alternative future materials for water purification?. Coord. Chem. Rev..

[14] Ghoraba Z., Aibaghi B., Soleymanpour A. (2017). Application of cation-modified sulfur nanoparticles as an efficient sorbent for separation and preconcentration of carbamazepine in biological and pharmaceutical samples prior to its determination by high-performance liquid chromatography. J. Chromatogr. B Anal. Technol. Biomed. Life Sci..

[15] de Fátima Alpendurada M. (2000). Solid-phase microextraction: A promising technique for sample preparation in environmental analysis. J. Chromatogr. A.

[16] Eskandarloo H., Godec M., Arshadi M., Padilla-Zakour O.I., Abbaspourrad A. (2018). Multi-porous quaternized chitosan/polystyrene microbeads for scalable, efficient heparin recovery. Chem. Eng. J..

[17] Arshadi M., Eskandarloo H., Enayati M., Godec M., Abbaspourrad A. (2018). Highly water-dispersible and antibacterial magnetic clay nanotubes functionalized with polyelectrolyte brushes: High adsorption capacity and selectivity toward heparin in batch and continuous system. Green Chem..

[18] Enayati M., Abdolmaleki M.K., Abbaspourrad A. (2020). Synthesis of cross-linked spherical polycationic adsorbents for enhanced heparin recovery. ACS Biomater. Sci. Eng..

[19] Eskandarloo H., Arshadi M., Enayati M., Abbaspourrad A. (2018). Highly efficient recovery of heparin using a green and low-cost quaternary ammonium functionalized halloysite nanotube. ACS Sustain. Chem. Eng..

[20] Zhou H.-C., Long J.R., Yaghi O.M. (2012). Introduction to metal–organic frameworks. Chem. Rev..

[21] Aunan E., Affolter C.W., Olsbye U., Lillerud K.P. (2021). Modulation of the thermochemical stability and adsorptive properties of MOF-808 by the selection of non-structural ligands. Chem. Mater..

[22] Luo Y., Estudillo-Wong L.A., Cavillo L., Granozzi G., Alonso-Vante N. (2016). An easy and cheap chemical route using a MOF precursor to prepare Pd–Cu electrocatalyst for efficient energy conversion cathodes. J. Catal..

[23] Wang C., Kim J., Tang J., Na J., Kang Y., Kim M., Lim H., Bando Y., Li J., Yamauchi Y. (2020). Large-scale synthesis of MOF-derived superporous carbon aerogels with extraordinary adsorption capacity for organic solvents. Angew. Chem. Int. Ed..

[24] Faust T. (2016). MOFs move to market. Nat. Chem..

[25] Rubio-Martinez M., Avci-Camur C., Thornton A.W., Imaz I., Maspoch D., Hill M.R. (2017). New synthetic routes towards MOF production at scale. Chem. Soc. Rev..

[26] Ryu U., Jee S., Rao P.C., Shin J., Ko C., Yoon M., Park K.S., Choi K.M. (2021). Recent advances in process engineering and upcoming applications of metal–organic frameworks. Coord. Chem. Rev..

[27] Chen B., Yang Z., Zhu Y., Xia Y. (2014). Zeolitic imidazolate framework materials: Recent progress in synthesis and applications. J. Mater. Chem. A.

[28] Jiao C., Li M., Ma R., Wang C., Wu Q., Wang Z. (2016). Preparation of a Co-doped hierarchically porous carbon from Co/Zn-ZIF: An efficient adsorbent for the extraction of trizine herbicides from environment water and white gourd samples. Talanta.

[29] Liang C., Zhang X., Feng P., Chai H., Huang Y. (2018). ZIF-67 derived hollow cobalt sulfide as superior adsorbent for effective adsorption removal of ciprofloxacin antibiotics. Chem. Eng. J..

[30] Ganta D., Guzman C., Combrink K., Fuentes M. (2021). Adsorption and Removal of Thymol from Water Using a Zeolite Imidazolate Framework-8 Nanomaterial. Anal. Lett..

[31] Bayati B., Ghorbani A., Ghasemzadeh K., Iulianelli A., Basile A. (2019). Study on the separation of H_2_ from CO_2_ using a ZIF-8 membrane by molecular simulation and Maxwell-Stefan model. Molecules.

[32] Sahoo T.R., Prelot B., Bonelli B., Freyria F.S., Rossetti I., Sethi R. (2020). Adsorption processes for the removal of contaminants from wastewater: The perspective role of nanomaterials and nanotechnology. Micro and Nano Technologies, Nanomaterials for the Detection and Removal of Wastewater Pollutant.

[33] Shokrollahi A., Alizadeh A., Malekhosseini Z., Ranjbar M. (2011). Removal of bromocresol green from aqueous solution via adsorption on *Ziziphus nummularia* as a new, natural, and low-cost adsorbent: Kinetic and thermodynamic study of removal process. J. Chem. Eng. Data.

[34] Ghadim E.E., Manouchehri F., Soleimani G., Hosseini H., Kimiagar S., Nafisi S. (2013). Adsorption properties of tetracycline onto graphene oxide: Equilibrium, kinetic and thermodynamic studies. PLoS ONE.

[35] Ghaedi M., Shokrollahi A., Hossainian H., Kokhdan S.N. (2011). Comparison of activated carbon and multiwalled carbon nanotubes for efficient removal of eriochrome cyanine R (ECR): Kinetic, isotherm, and thermodynamic study of the removal process. J. Chem. Eng. Data.

[36] Ho Y.S., McKay G. (1999). Pseudo-second order model for sorption processes. Process Biochem..

[37] Simonin J.P. (2016). On the comparison of pseudo-first order and pseudo-second order rate laws in the modeling of adsorption kinetics. Chem. Eng. J..

[38] Robati D. (2013). Pseudo-second-order kinetic equations for modeling adsorption systems for removal of lead ions using multi-walled carbon nanotube. J. Nanostruct. Chem..

[39] Azizi S., Shahri M.M., Mohamad R. (2017). Green synthesis of Zinc oxide nanoparticles for enhanced adsorption of lead ions from aqueous solutions: Equilibrium, kinetic and thermodynamic studies. Molecules.

[40] Wu F.-C., Tseng R.-L., Juang R.-S. (2009). Initial behavior of intraparticle diffusion model used in the description of adsorption kinetics. Chem. Eng. J..

[41] Chien S.H., Clayton W.R. (1980). Application of Elovich equation to the kinetics of phosphate release and sorption in soils. Soil Sci. Soc. Am. J..

